# Early Cerebral Microvasculature Impairment and Increased Body Mass Index in Patients with Psoriasis

**DOI:** 10.3390/biomedicines12081627

**Published:** 2024-07-23

**Authors:** Katarzyna Piec, Luiza Marek-Józefowicz, Katarzyna Nadolska, Adam Lemanowicz, Zbigniew Serafin, Grzegorz Kozera

**Affiliations:** 1 Department of Neurology, Faculty of Medicine, Ludwik Rydygier Medical College in Bydgoszcz, Nicolaus Copernicus University in Torun, 87-100 Torun, Poland; katarzyna.piec@cm.umk.pl; 2 Department of Dermatology and Venereology, Faculty of Medicine, Ludwik Rydygier Medical College in Bydgoszcz, Nicolaus Copernicus University in Torun, 87-100 Torun, Poland; 3 Department of Radiology, Faculty of Medicine, Ludwik Rydygier Medical College in Bydgoszcz, Nicolaus Copernicus University in Torun, 87-100 Torun, Polandadam.lemanowicz@cm.umk.pl (A.L.); serafin@cm.umk.pl (Z.S.); 4 Faculty of Medical Sciences, Bydgoszcz University of Science and Technology, 85-796 Bydgoszcz, Poland; 5 Centre of Medical Simulations, Faculty of Medicine, Medical University of Gdansk, 80-210 Gdansk, Poland

**Keywords:** cerebral autoregulation, obesity, BMI, psoriasis, Transcranial Doppler

## Abstract

Psoriasis induces systemic atherosclerosis, but its impact on cerebrovascular function remains unclear. However, stroke prevention must be considered in psoriasis, as it is commonly comorbid with classic cardiovascular risk factors. Thus, the aim of the study is to assess cerebral microvasculature function and its confounders in patients with psoriasis. The study protocol included cerebral autoregulation assessment with measurements of vasomotor reactivity reserve (VMRr) on the middle cerebral arteries with the use of a Transcranial Doppler (TCD) in 50 patients with psoriasis without cerebrovascular events (46; 21–74 years) and 26 healthy controls (41; 29–58 years). Analyses of VMRr relationships with the psoriasis course, comorbidities, inflammatory markers and intima–media thickness (IMT) were performed. The study showed that VMRr was lower (64% vs. 76%, *p* = 0.001), and the IMT was higher (0.65 vs. 0.52 mm, *p* = 0.001) in patients compared to controls. The patients were also characterized by a higher body mass index (BMI) and a higher level of Il-6 than the controls (29.14 vs. 25.76 kg/m^2^, *p* = 0.004 and 585 vs. 204 pg/mL, *p* < 0.001, respectively), but only BMI was independently impacting VMRr reduction (*p* = 0.02). In conclusion, early cerebral microvasculature dysfunction may occur in patients with psoriasis, and its extent is associated with an increase in BMI. Thus, body mass reduction should be strongly recommended for stroke prophylaxis in patients with psoriasis.

## 1. Introduction

The burden of cerebrovascular diseases (CVDs) remains one of the most crucial epidemiological, medical and economic challenges for healthcare systems in the 21st century [1,2,3]. It is associated with a heterogeneity of causes, including the presence of both cerebral microangiopathy and macroangiopathy [4,5,6]. Apart from numerous classical vascular factors underlying the majority of CVD events, an increasing number of novel non-classical risk factors, including obesity, lack of physical activity, environmental conditions and chronic inflammatory status, are currently being described [3]. In particular, the influence of systemic inflammatory diseases on the development and course of CVDs has been widely proven [6,7,8].

Psoriasis is chronic inflammatory dermatosis with numerous comorbidities and systemic consequences, including a higher presence of overweight and obesity [9,10,11,12,13,14,15,16]. The understanding of its pathogenesis has changed dramatically over the past two decades, and attention is being paid to vascular injury in the course of this dermatosis [17,18,19].

The common pathophysiological determinants of psoriasis and CVD are based mainly on Th1 and Th17 lymphocytes, which influence inflammation and angiogenesis [20]. Patients suffering from both psoriasis and CVD have increased serum levels of Th17-dependent cytokines IL-6, IL-21, IL-22 and TNF. The presence of these cytokines leads to dysfunction of the vascular endothelium and, through its stimulating effect on the production of prothrombotic factors, increases the risk of CVD [21].

Because patients with psoriasis are characterized by increased vascular stiffness, reduced vascular contractility or flexibility, and a thickening of the intima–media in carotid arteries, psoriasis can be regarded as an independent risk factor for both systemic and cerebral macrovascular injury [22,23]. Theoretically, the presence of endothelial dysfunction observed in this dermatosis might also induce cerebral microvascular damage, including both preclinical functional impairment and overt small vessel disease [24,25]; however, such associations have not been evidenced to date [26].

Cerebrovascular reactivity (CVR) impairment reflects the dysfunction of the cerebral microvasculature. Previous reports based on CVR testing with the use of TCD evidenced the presence of the early impairment of cerebral microcirculation in patients with chronic diseases with inflammatory manifestation [27,28,29,30], including components of metabolic syndrome such as obesity or overweight [31,32]. Consequently, CVR reduction has been found to be associated with both silent white matter lesions [33], overt lacunar infarctions [34], carotid artery occlusion [35] and migraine [36]. Interestingly, the alteration of CVR parameters in psoriasis has not been studied so far. Nevertheless, evidence for reduced CVR in psoriasis patients without clinically overt CVD could additionally confirm a beneficial role of strict control of modifiable risk factors for primary CVD prevention in patients with this dermatosis, as shown previously in patients with arterial hypertension or type 1 diabetes [6,28,29,33,34].

Therefore, the aim of our study was to evaluate CVR in patients with psoriasis without a prior history of CVD and to assess the relationship between CVR and psoriasis co-morbidities and the presence of macroangiopathic injury.

## 2. Materials and Methods

### 2.1. Patients and Controls

The study population consisted of 50 Caucasian adults with psoriasis (19 women and 31 men, median age 46, 21–74 years) treated and consecutively recruited in the Department of Dermatology and Venereology at Ludwik Rydygier Medical College in Bydgoszcz. The psoriasis duration ranged from 0.5 to 34 years, the diagnosis was established between 5 and 64 years of age, and the mean Psoriasis Area and Severity Index (PASI) was 19.11 (±5.83). Concomitant risk factors included overweight or obesity (in 82% of patients), hyperlipidemia (in 76%), arterial hypertension (in 46%), type 2 diabetes (in 14%), and active cigarette smoking (in 14%). The control group consisted of 26 non-smoking healthy volunteers (13 women and 13 men, median age 41 years, range limits 29 to 58), living in the same region as the study group, having similar education (measured in years of education), without diagnosed hypertension, diabetes, or hyperlipidemia and not taking medication for chronic diseases, with a negative family history of psoriasis and other chronic skin diseases.

Patients with recent systemic therapies (methotrexate, cyclosporin, biologics, phototherapy or photochemotherapy within the preceding 3 months) or topical treatment of psoriasis (corticosteroids within 2 weeks) were excluded from the study. We also excluded patients and controls with neurological deficits, history of head trauma or previous cerebrovascular events (stroke or transient ischemic attack) or presence of stroke foci on magnetic resonance imaging (1.5 T unit: Optima 450w GEM, GE Healthcare, Milwaukee, WI, USA), hemodynamically significant (>50% of the vessel lumen) stenosis of the extracranial arteries and alcohol or drug addiction. Additional exclusion criteria for all participants included a lack of an effective bone window for TCD examination.

All the tests were performed in standardized conditions of lighting, humidity and silence in the room at the same time of the day. The study protocol included history taking, neurological examination, measurement of BMI and resting values of blood pressure and heart rate, laboratory testing, extracranial ultrasound with IMT assessment and TCD with measurement of the vasomotor reserve. 

The study protocol was approved by the Medical Ethics Committee of The Nicolaus Copernicus University in Toruń (KB 695/2017). The study was conducted between November 2017 and December 2020. Upon entry, each participant gave her/his informed consent. All methods were performed in accordance with the relevant guidelines and regulations (Declaration of Helsinki).

### 2.2. Subjects’ Characteristics

Each patient’s history was obtained, including information on past and current disorders as well as on co-morbid conditions. Weight and height were recorded and expressed as BMI. Neurological deficits were excluded by neurological examination performed by a certified neurologist. Each patient also underwent an examination of the smooth and hairy skin carried out by a dermatologist, where the severity of psoriasis was assessed by calculating the PASI index (percentage of the skin area covered by psoriatic lesions) [37]. Laboratory examinations in patients with psoriasis included measurements of interleukin-6 (IL-6) and lipid profile. To determine the concentration of IL-6, the ELISA (Qayee-Bio) method was used. The examined biological material was serum. A double-antibody sandwich enzyme-linked immunosorbent one-step process to assess the level of IL-6 was applied. The test range was 15.6 pg/mL–1000 pg/mL. In the final result, OD at 450 nm wavelength was measured.

Hypertension was diagnosed if two consecutive measurements of the systolic and diastolic blood pressures exceeded 140 and 90 mmHg, respectively, or if antihypertensive medication was used [38]. Hyperlipidemia was diagnosed if the low-density lipoprotein cholesterol (LDL-C) and/or triglycerides exceeded 115 and 150 mg/dL, respectively, or if cholesterol/triglyceride-lowering medications were used [39]. 

### 2.3. Intima–Media Thickness Measurement

The IMT in both carotid arteries was measured with the B-mode ultrasound examination of the carotid arteries/carotid ultrasound/using an ESAOTE ultrasound machine equipped with a linear probe with a central working frequency of 7.5 MHz and range limits of 5–10 MHz after standard examination of both carotid arteries. During each examination, the distal 2 cm long segment of the common carotid artery in two different (anterior–posterior and lateral) longitudinal projections was assessed bilaterally. The final value of the IMT was calculated as a mean of four measurements (two projections, both sides). The IMT was measured using a semiautomatic method (Carotid Measure System) with regard to the Mannheim Consensus criteria [40].

### 2.4. Transcranial Doppler Examination

**Figure 1:**
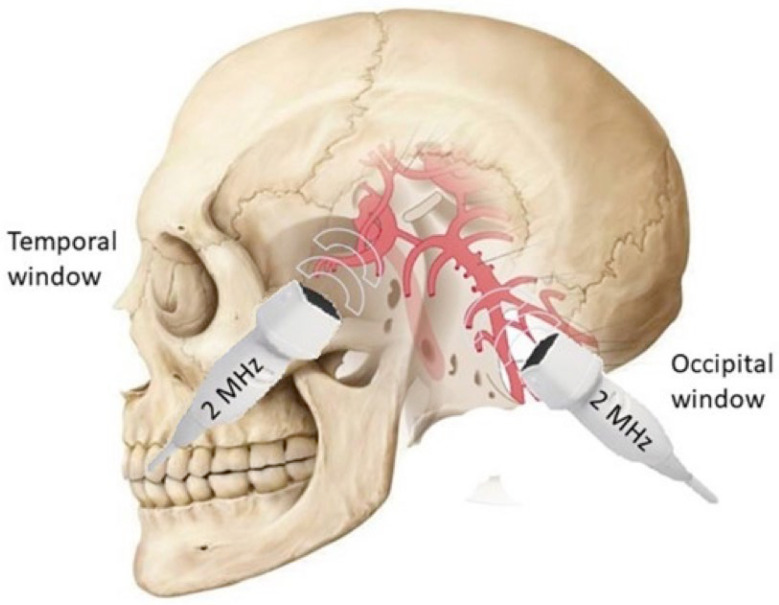
Bone windows typically used in TCD examination.

TCD is an appreciated tool for assessing cerebral circulation, allowing for the assessment of cerebral blood flow (CBF) velocity and flow direction in the main cerebral vessels (circle of Willis) using the Doppler effect [41]. CBF velocity measurement using TCD has a significant advantage over other methods (such as single-photon emission tomography (SPECT), positron emission tomography (PET) or magnetic resonance imaging (MRI)) due to the ability to use noninvasive continuous signal analysis. Due to the high scattering of ultrasound by the skull bones, during the transcranial examination, a 2 MHz probe is usually applied to the so-called “bone windows”—areas of natural cranial bone thinning in the temporal area (temporal windows) or natural openings of the skull (suboccipital and transorbital windows) (Figure 1). A limitation of the TCD method may be the lack of a temporal bone window, which prevents the assessment of flow in the arteries of the circle of Willis in 10–20% of the population, more often in postmenopausal women. 

**Figure 2:**
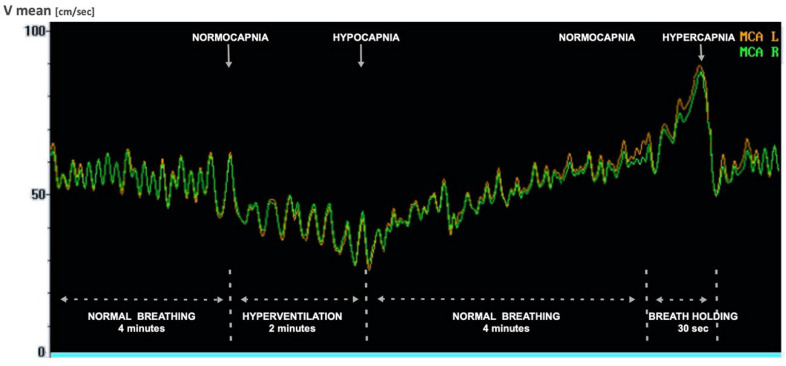
TCD recording of flow velocity in the MCA during hyperventilation and breath-holding tests. Abbreviations: V mean = mean velocity, MCA L = left middle cerebral artery, MCA R = right middle cerebral artery.

Vessel identification in TCD is based on the location and direction of the probe application, measurement depth, blood flow direction, Doppler spectrum shape and the use of activation tests. Unfortunately, the low spatial resolution of TCD enables only a direct assessment of flow parameters in large cerebral arteries (e.g., middle cerebral artery, MCA). However, TCD may also assess cerebral microcirculation indirectly, e.g., with testing of cerebral autoregulation. Vasomotor reactivity measurement is one of the techniques for cerebral autoregulation assessment, based on the ability to change the diameter of the small brain vessels in response to a vasoactive substance, most often CO_2_. Hypercapnia causes dilation of cerebral microvessels and consequently increases the flow velocity in supplying large vessels, while hypocapnia causes a decrease in the diameter of small vessels and secondarily reduces flow velocity in supplying arteries of the circle of Willis. The vasomotor reactivity reserve of an artery is the range between the maximum and minimum flow caused by changes in CO_2_ pressure (Figure 2). 

In our research, MCA flow parameters were measured by using a Multi Dop and Viasys Sonara Companion III device ultrasound machine equipped with dedicated software. Velocity measurements were performed simultaneously in both MCAs with the use of a two-channel monitoring kit: two probes with a 2 MHz pulse wave, the fixation band and the monitoring program. The physiological techniques of provoking cerebrovascular reactivity by changes in partial CO_2_ concentrations (pCO_2_) were applied according to the published standards [42,43]. Hyperventilation, i.e., 2 min of deep breathing test, was used to induce hypocapnia, which resulted in minimization of CBF after about 30 s. Maximization of the flow accompanying hypercapnia was maintained using 30 s of breath holding. During the CO_2_ reactivity test, the end-tidal CO_2_ concentration in the expired air was monitored continuously by capnograph. After both hyperventilation and breath holding, significant (*p* < 0.01) changes in the pCO_2_ were obtained. VMRr, expressed in percent change from the baseline, was calculated according to the standard protocol published previously [42,43]. The median values of VMRr obtained from both MCAs were used for further analyses. Due to the unilateral lack of a bone window in 14 patients, the parameters were determined only for the contralateral MCA. In other cases, the measurement value obtained for the left and right arteries was averaged. The VMRr value was calculated using the following formula:

VMRr = (max V mean − min V mean/rest V mean) × 100% (1)

(rest V mean—average resting velocity in MCA; max V mean—average maximum velocity in MCA during hypercapnia; min V mean—average minimum velocity in MCA during hypocapnia).

### 2.5. Reproducibility

The reproducibility of the measurements of the VMRr was assessed in a group of 8 healthy volunteers, based on three consecutive measurements performed according to the study protocol, for both middle cerebral arteries. There was no statistically significant difference between the mean values of the VMRr in the individual measurement cycles. The differences between the average values of the VMRr determined for each measurement cycle and the average value of VMRr from all three measurement cycles did not exceed 2.5%. The unbiased intra-class correlation coefficient (ICC) was 0.87.

### 2.6. Statistics

Statistical analysis was performed using the Statistica 13.3 program (TIBCO Software Inc., Palo Alto, CA, USA (2017) Statistica (data analysis software system), version 13. https://www.statsoft.pl/statistica_13_3/). Descriptive statistics (mean, standard deviation, median, minimum and maximum values) were calculated for all variables. The agreement of the data with the normal distribution was assessed using the W Shapiro–Wilk test. Intergroup relationships were compared using independent-sample tests: Student’s *t*-test with independent variance assessment and the Mann–Whitney U, ANOVA and ANCOVA tests. The relationships between categorized variables were assessed with the chi-squared test. Multivariate regression models were used to assess independent factors affecting the VMRr and IMT. The level of *p* < 0.05 was regarded as statistically significant.

## 3. Results

Patients with psoriasis had lower VMRr compared to controls and higher average BMI, obesity rates, blood pressure values (systolic, diastolic and average) and IL-6 levels when compared to the controls. There were no other differences in age, gender and heart rate or the proportion of patients with BMI > 25 kg/m^2^ between those groups (Table 1). 

Comparisons between patients (*n* = 7) and controls (*n* = 9) with BMI < 25 kg/m^2^ showed a higher mean of IL-6 in patients than in controls (543 pg/mL, 281–627 vs. 216 pg/mL, 124–321; *p* < 0.01); no differences regarding VMRr (0.74%, SD ± 0.13 vs. 0.70%, SD ± 0.12; *p* = 0.57) or IMT (0.54 mm, 0.46–0.90 vs. 0.48 mm, 0.38–0.99; *p* = 0.24) existed. There was a correlation between BMI and IL-6 in the whole group that included both patients and controls (r = 0.32, *p* < 0.01) but not in the subgroup of participants with BMI < 25 kg/m^2^ (r = 0.21, *p* = 0.42). Multivariate regression analysis showed that psoriasis was the only independent factor influencing the reduction of VMRr in the entire group, including both patients and controls (beta −0.01, *p* = 0.03); no significant effect of hypertension, diabetes, smoking or statin use existed.

**Figure 3:**
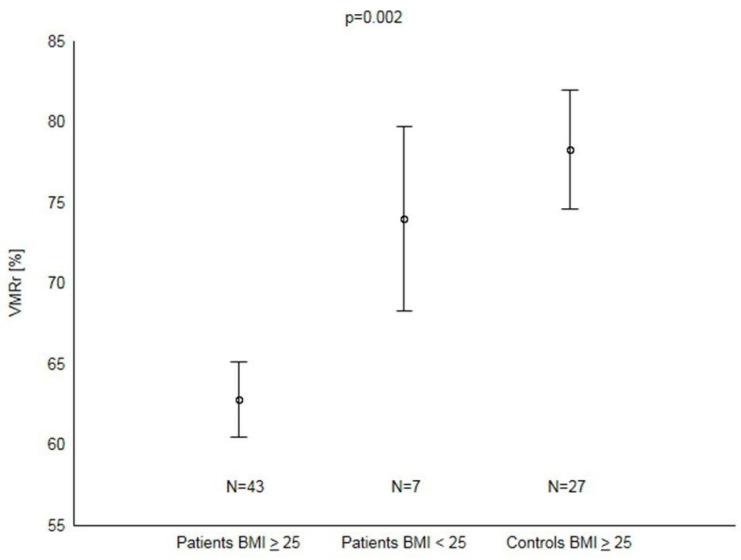
VMRr in patients with psoriasis and controls with an increased BMI. Abbreviations: VMRr = vasomotor reactivity reserve; BMI = body mass index; *n* = number of patients.

The significant decrease in VMRr occurred in patients with psoriasis and BMI ≥ 25 kg/m^2^ when compared to controls with BMI ≥ 25 kg/m^2^ (*p* < 0.01). There was no significant difference between patients with BMI < 25 kg/m^2^ and controls (*p* = 0.52) or subgroups of patients with normal and abnormal BMI (*p* = 0.07) (Figure 3). There were no differences in VMRr between specific patient subgroups based on the presence or absence of comorbidities or their specific treatments (Table 2). In the group of psoriasis patients, a negative correlation was found between VMRr and Il-6 or BMI (Table 3). No significant correlation between PASI and BMI (r = 0.08, *p* = 0.57) or IL-6 (r = 0.03, *p* = 0.81) existed. Multivariate regression analysis showed that the BMI was the only independent factor affecting VMRr in patients with psoriasis (Table 4).

**Figure 4:**
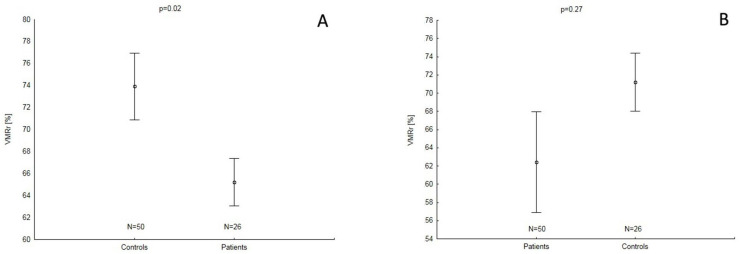
VMRr in patients with psoriasis and controls covariated with BMI (mean of BMI 27.98 kgm^2^) (**A**) and covariated with IL-6 (median of IL-G 454.74 pg/mL) (**B**). Abbreviations: VMRr = vasomotor reactivity reserve; BMI = body mass index; IL-6 = interleukin-6; *n* = number of patients.

The study also showed that the IMT value was higher in patients with psoriasis than in the controls (Table 1). However, VMRr impairment existed only after controlling for BMI but not for IL-6 (Figure 4A,B). In the patients’ group, the IMT was higher in subjects with arterial hypertension or in those on ACE/ARB treatment and was correlated with the patient’s age and age of onset of psoriasis (Table 2 and Table 3). No correlation between VMRr and IMT existed in either the patients (r = −0.07; *p* = 0.72) or the controls (r = 0.03; *p* = 0.65), and the patient’s age was the only independent factor for IMT (Table 4).

## 4. Discussion

Our study revealed the presence of impaired cerebral microcirculation in patients with psoriasis who did not have overt cerebrovascular events or neurological deficits. The reduction in VMRr was independently influenced by patient BMI but not by IL-6 or IMT. The reduction in VMRr was independently influenced by patient BMI but not by inflammatory markers or IMT. The use of the assessment of vasomotor reactivity of cerebral vessels using TCD in patients with psoriasis in our study is pioneering because, to the best of our knowledge, there are no publications in literature so far using this parameter to detect microangiopathy in patients with this dermatosis. There are only a few reports on microangiopathy assessed by neuroimaging, mainly magnetic resonance imaging of the brain in patients with psoriasis [26], but there is no information about microcirculatory dysfunction in these patients. 

Our results indicate a significant impact of increased BMI on the occurrence and degree of cerebral autoregulation disorders. Although there are no previous reports on the impact of obesity on cerebral microcirculation in patients with psoriasis, our results are consistent with the study published by Rodríguez-Flores et al. [44], who found that increased BMI is an independent factor of CVR impairment in patients with insulin resistance. Reduced CBF associated with obesity or overweight has also been demonstrated using non-TCD methods, such as spin labeling MRI [32,45], and was ultimately confirmed in a single meta-analysis [31].

Interestingly, a significant reduction in VMRr in patients with psoriasis and BMI ≥ 25 kg/m^2^ compared to the control group without psoriasis and BMI ≥ 25 kg/m^2^ indicates an additional and significant negative impact of increased BMI on the autoregulation of CBF in patients with psoriasis, based on the “second hit” phenomenon. Our study, as evidenced by many previous studies [46], showed a higher rate of obesity among patients suffering from psoriasis [12,47,48,49]. 

Studies conducted so far that assess the effect of excess body weight on the structural image of the brain, mainly using MRI, have shown a statistically significant increase in abnormalities compared to the control group. A decrease in brain volume, both gray and white matter, was associated with the development of cerebral small vessel disease or the presence of neurodegenerative diseases [50]. Interestingly, it was shown that the genetic predisposition to a higher BMI itself did not increase the risk of cerebrovascular diseases, and only acquired obesity, especially that expressed by the waist-to-hip ratio, influenced the frequent occurrence of various phenotypes of cerebrovascular diseases [51]. This conclusion, in accordance with our studies, emphasizes the important role of maintaining a healthy body weight, especially in patients with additional disease burdens (such as psoriasis) in CVD prevention.

Consistent with previous literature reports, we found higher IMT values in patients compared to controls [23,24]. Oxidative stress and inflammation are recognized as an initial step in the pathophysiological cascade of psoriasis, which leads to endothelial dysfunction. In psoriasis and atherosclerosis, similar immune pathways are activated. The same pro-inflammatory cytokines have been proven to be involved (e.g., tumor necrosis factor (TNF-α), interferon-gamma (IFN-γ), IL-2, IL-6, IL-8) both in the formation of atherosclerotic plaque in the blood vessel and psoriatic plaque in the skin. Patients with psoriasis are characterized by thickening of the intima–media complex, vascular stiffness, impaired vascular contractility and reduced elasticity. It is, therefore, worth emphasizing the important role of the potential therapeutic strategy of using antioxidants in the treatment of this dermatosis [52].

Many studies have shown a positive correlation between obesity and IMT [53,54], but our study did not show a statistically significant relationship. Based on backward stepwise analysis, we observed that age is an independent factor influencing IMT in people with psoriasis, which is consistent with previous data from the literature [55,56]. However, contrary to other reports, we did not find any association between IMT and PASI [55,57,58,59] or the duration of psoriasis [55]. We also found no relationship between vasomotor reactivity parameters and IMT in the group of patients and the control group. Indeed, similar results have been previously reported in diabetic patients [28]. 

The influence of chronic additional diseases, especially CVD, on the increase in inflammatory markers in patients with psoriasis is widely known. The systemic nature of this dermatosis is reflected in vascular complications—chronic inflammation occurring in psoriasis leads to the development of pathological changes in small blood vessels (called microangiopathy) and, through the accelerated development of atherosclerosis, promotes the development of pathology of medium- and large-caliber vessels (i.e., macroangiopathy). Macroangiopathy developing in both extracranial and intracranial vessels and microangiopathy of cerebral vessels, through disruption of CBF, increase the risk of stroke incidents and small vessel disease. Recent studies have confirmed a positive correlation between elevated inflammatory markers (e.g., higher neutrophil-to-lymphocyte ratio (NLR) and IL-22) in patients with psoriasis in specific age groups, as well as a higher pro-inflammatory state (i.e., as reflected by platelet indexes) in patients with psoriasis with comorbidities compared to people with this dermatosis without other chronic disease [60]. A significant influence of inflammation in the coexistence of additional disease burdens, including primarily obesity, was also demonstrated in the authors’ work. This knowledge is particularly important due to the possibility of faster implementation of treatment for additional diseases and the spread of prophylaxis of cardiovascular events in patients with psoriasis. Both psoriasis and obesity induce the production of inflammatory markers and mediators, such as TNF-α, and produce pro-inflammatory cytokines, including C-reactive protein and IL-6 [7,51]. The level of IL-6 shows a positive correlation with BMI and participates in the regulation of lipid metabolism, which is the basis of the pathogenesis of atherosclerosis [61]. It has been shown that inflammation associated with obesity can lead to a gradual increase in the risk of stroke, as increased IL-6 concentration in plasma is an important risk factor for CVD [62]. Several studies have also shown an association between elevated IL-6 levels and the severity of psoriasis [63,64]. Similarly, we have demonstrated higher serum IL-6 levels in psoriasis patients than in control subjects. Furthermore, we found a negative correlation between IL-6 and the extent of cerebral vasomotor reactivity. The lack of a significant effect of IL-6 on VMRr reduction in multivariate analyses may be partially explained by type 2 error due to the small sample size. Regardless, this indicates a potential effect of IL-6 on cerebral microcirculation. In our opinion, this issue requires the continuation of research on a larger sample because IL-6 stimulates the production of prothrombotic factors and increases the risk of CVD [65,66,67,68]. There are no publications yet on the effect of IL-6 on cerebral vessels in patients with psoriasis.

The single-center nature of the study, which may limit the generalizability of the results, as well as its cross-sectional nature, is an important limitation of our study. A small sample size may also reduce its potential to confirm some relationships. Nevertheless, we were able to show significant differences in both groups regarding the VMRr, BMI, IMT and IL-6. We also did not assess the radiological associates of cerebral microangiopathy. This undoubtedly interesting issue is a goal of our future study. Broadening the inflammatory markers that were tested was beyond the study’s available finances. Finally, an observational character of the research protocol may also be mentioned among its limitations. 

In summary, we conclude that adult patients with psoriasis may develop an early presence of cerebral microangiopathy, the severity of which is related to an increase in body mass index. Thus, the maintenance of a normal BMI should be strongly recommended for early stroke prevention in those patients.

## Figures and Tables

**Table 1:** Characteristics of the study population of patients with psoriasis and healthy controls.

	Patients with Psoriasis	Healthy Control Subjects	*p*
Number of study participants	50	26	
Age [years] (median, range)	46 (21–74)	41 (29–58)	0.062
Male [*n*/%]	19/38	13/50	0.316
VMRr [%](mean, SD)	64 (SD 0.17)	76 (SD 0.11)	0.001
IMT [mm] (median, range)	0.65 (0.42–1.03)	0.52 (0.37–0.99)	0.001
BMI [kg/m^2^] (median, range)	29 (18–41)	26 (19–32)	0.004
BMI > 25 kg/m^2^ [*n*/%]	43/86	17/58	0.106
BMI > 30 kg/m^2^ [*n*/%]	20/40	2/8	0.007
Il-6 [pg/mL] (median, range)	585 (281–782)	204 (97–369)	<0.001
Hypertension [*n*/%]	23/46	-	-
ACEI treatment [*n*/%]	16/32	-	-
Hyperlipidemia [*n*/%]	38/76	-	-
Statin treatment [*n*/%]	6/12	-	-
Diabetes [*n*/%]	7/14	-	-
Smoking [*n*/%]	7/14	-	-
Psoriasis arthritis [*n*/%]	9/18	-	-
Onset < 18 year [*n*/%]	11	-	-
PASI > 20 points [*n*/%]	22	-	-
PASI [points] (median, range)	18.1 (12.0–33.8)	-	-
Duration of psoriasis [years] (median, range)	14.5 (0.5–34.0)	-	-
Duration of last psoriasis exacerbation [weeks] (median, range)	6.0 (1.0–16.0)	-	-

Abbreviations: BMI = body mass index; VMRr = vasomotor reactivity reserve; Il-6 = interleukin-6; IMT = intima–media thickness; ACEI = angiotensin-converting enzyme inhibitor; PASI = Psoriasis Area Severity Index.

**Table 2:** Impact of vascular risk factors and comorbidities on VMRr and IMT in patients with psoriasis.

Co-Morbid Condition	*n*	VMRr			IMT		
		Present	Absent	*p*	Present	Absent	*p*
Hyperlipidemia	38	0.63	0.69	0.296	0.66	0.64	0.690
Arterial hypertension	23	0.62	0.66	0.409	0.74	0.58	<0.001
ACEI treatment	16	0.60	0.67	0.170	0.76	0.60	0.001
Statin treatment	6	0.65	0.64	0.976	0.76	0.64	0.092
Diabetes	7	0.58	0.65	0.309	0.72	0.64	0.259
Smoking	7	0.7	0.64	0.382	0.71	0.64	0.259
Psoriasis arthritis	9	0.7	0.63	0.290	0.61	0.66	0.245
PASI > 20 points	22	0.64	0.64	0.989	0.67	0.64	0.616
Onset < 18 year	11	0.67	0.64	0.536	0.59	0.67	0.150
Male	31	0.64	0.65	0.734	0.67	0.63	0.348
Systemic treatment	30	0.65	0.63	0.712	0.65	0.65	0.922

Abbreviations: *n* = amount of psoriasis patients with co-morbid condition; VMRr = vasomotor reactivity reserve; IMT = intima–media thickness; ACEI = angiotensin-converting enzyme inhibitor; PASI = Psoriasis Area Severity Index.

**Table 3:** Correlations between the VMRr and IMT with age, BMI, average blood pressure and heart rate, inflammatory markers and the clinical course of psoriasis.

	VMRr		IMT	
	R	*p*	R	*p*
Age	−0.07	0.622	0.66	0.001
BMI	−0.40	0.004	0.13	0.366
Average blood pressure	−0.16	0.275	0.13	0.378
Heart rate	−0.09	0.551	0.01	0.958
Il-6	−0.35	0.014	−0.19	0.192
Age of onset	0.01	0.995	0.51	0.001
Duration of psoriasis (years)	−0.12	0.417	0.09	0.529
Duration of last psoriasis exacerbation (weeks)	0.11	0.466	0.18	0.201
PASI (points)	−0.02	0.86	−0.02	0.87

Abbreviations: BMI = body mass index; VMRr = vasomotor reactivity reserve; Il-6 = interleukin-6; IMT = intima–media thickness; PASI = Psoriasis Area Severity Index.

**Table 4:** Results of multivariable linear regression analyses performed in a group of patients with psoriasis to assess VMRr and IMT.

	VMRr		IMT	
	Beta	*p*	Beta	*p*
BMI	−0.33	0.02	n/a	n/a
IL-6	−0.25	0.06	n/a	n/a
Arterial hypertension	n/a	n/a	0.22	0.12
Statin treatment	n/a	n/a	−0.06	0.61
Age [years]	n/a	n/a	0.55	<0.01

Abbreviations: BMI = body mass index; VMRr = vasomotor reactivity reserve; IMT = intima–media thickness; IL-6 = interleukin-6.

## Data Availability

The data presented in this study are available on request from the corresponding and first author (G.K. and K.P.).

## Abbreviations

BMI	Body mass index
VMRr	Vasomotor reactivity reserve
IL-6	Interleukin-6
IMT	Intima–media thickness
CVD	Cerebrovascular disease
CBF	Cerebral blood flow
CVR	Cerebrovascular reactivity
TCD	Transcranial Doppler
PASI	Psoriasis Area Severity Index
MCA	Middle cerebral artery
pCO2	Partial CO_2_ concentration
ICC	Intra-class correlation coefficient
ACEI	Angiotensin-converting enzyme inhibitor
MRI	Magnetic resonance imaging
